# Disseminated Tuberculosis Complicated by Pulmonary Thromboembolism

**DOI:** 10.7759/cureus.89662

**Published:** 2025-08-08

**Authors:** Preethi S Suresh, Koushik Muthu Raja Mathivanan

**Affiliations:** 1 Pulmonary Medicine, Sri Ramachandra Medical College, Chennai, IND; 2 Respiratory Medicine, Sri Ramachandra Institute of Higher Education and Research, Chennai, IND

**Keywords:** antitubercular therapy, extrapulmonary tuberculosis, hypercoagulable state, mycobacterium tuberculosis, osteomyelitis, pulmonary thromboembolism, tuberculosis, tuberculous peritonitis

## Abstract

Tuberculosis (TB) is a multisystem infectious disease with both pulmonary and extrapulmonary manifestations. TB can also induce a hypercoagulable state, setting off a cascade of changes in the body, including systemic inflammation, endothelial dysfunction, and abnormalities in the coagulation and fibrinolytic pathways. Collectively, these factors significantly increase the risk of venous thromboembolism, such as deep vein thrombosis and pulmonary embolism. These complications can worsen the overall disease course and complicate TB management.

We report the case of a 27-year-old man presenting with high-grade fever, abdominal pain, and significant weight loss. Imaging suggested tuberculous peritonitis, and computed tomography pulmonary angiography revealed pulmonary thromboembolism, a rare but serious TB-associated complication. Omental biopsy confirmed TB based on histopathology and GeneXpert. Despite the initiation of anti-tubercular therapy and anticoagulation, the patient developed osteomyelitis of the right iliac bone with an intramuscular abscess, which tested positive for *Mycobacterium tuberculosis*. This case highlights the thrombotic and multisystemic nature of TB and the need for vigilance in identifying its uncommon complications.

## Introduction

Tuberculosis (TB), which is caused by *Mycobacterium tuberculosis*, remains a significant global health burden with various clinical manifestations depending on the organs involved. While pulmonary TB accounts for the majority of cases, extrapulmonary involvement can affect the gastrointestinal tract, genitourinary system, bones, lymph nodes, and central nervous system, especially in immunocompromised individuals [1].

Beyond its infectious pathology, TB is increasingly associated with a hypercoagulable state that predisposes individuals to venous thromboembolism, including deep vein thrombosis (DVT) and pulmonary thromboembolism (PTE). This prothrombotic tendency is driven by mechanisms such as thrombocytosis, elevated fibrinogen, reduced levels of antithrombin III and protein C, and endothelial dysfunction [1]. Inflammatory cytokines such as tumor necrosis factor alpha (TNF-α) and interleukin 6 (IL-6) further enhance coagulation by upregulating procoagulant pathways and suppressing fibrinolysis [2].

Although PTE is considered an uncommon complication of TB, research shows it can occur in about 3.4% of hospitalized TB patients. PTE in TB patients is often under-recognized because of overlapping symptoms such as breathlessness, pleuritic chest pain, cough, and hypoxemia [3]. 

Some patients present with de novo pulmonary embolism (DNPE) without detectable DVT. This form may have a more favorable prognosis than embolism associated with concurrent DVT [4]. By contrast, patients with both DVT and PTE often have a greater thrombotic burden and worse outcomes [4]. Given these risks, clinicians should maintain a high index of suspicion for thromboembolic events in TB patients, particularly those with disseminated disease or unexplained respiratory deterioration. Early diagnosis and timely initiation of anticoagulation are critical for improving outcomes.

## Case presentation

We present the case of a 27-year-old man from Chennai with no known comorbidities who came to the emergency department with complaints of high-grade fever and vomiting for two days, lower abdominal pain for three days, and loss of appetite, with weight loss of approximately 10 kg over two months. He denied any known contact with a TB patient.

He was spiking high-grade fever, with a temperature of 101°F. He had tachycardia, with a heart rate ranging from 100 to 110 beats per minute. His blood pressure was within normal limits. A chest examination revealed bilateral normal vesicular breath sounds.

Abdominal examination showed tenderness on palpation in the right iliac fossa. No palpable mass was noted. Bowel sounds were present. There were palpable, tender, and mobile bilateral inguinal lymph nodes. The sizes of the right and left inguinal nodes were approximately 2×2 cm and 1×2 cm, respectively.

Laboratory findings (Table 1) on admission showed mild leukocytosis with elevated ESR, CRP, and D-Dimer levels. Hemoglobin and platelet counts were within normal limits. Renal and liver function tests were also normal. 

**Table 1:** Laboratory workup The table summarizes laboratory investigations with reference values at admission. Significant findings include leukocytosis with elevated inflammatory markers.

Laboratory Parameters	Patient's Value	Reference Range
Complete Blood Counts (CBC)		
Hemoglobin	11	13–17 g/dl
Total counts	13,200	4000–11000 /mm³
Platelets	3.23	1.5 – 4.5 lakhs/mm³
Renal Function Test (RFT)		
Blood Urea Nitrogen	18	6 – 20 mg/dl
Creatinine	0.8	0.7 – 1.2 mg/dl
Liver Function Test (LFT)		
SGOT	20	< 40 IU/L
SGPT	32	< 41 IU/L
ALP	98	32–120 IU/L
Total Protein	5.5	6.6 – 8.7 g/dl
Albumin	3.2	3.5 – 5.2 g/dl
Globulin	1.5	2 – 3.5 g/dl
A:G ratio	3.4	1.1 – 2.0
Total Bilirubin	0.28	< 1.2 mg/dl
Direct Bilirubin	0.15	< 0.30
Indirect Bilirubin	0.13	0.1 – 1.0
Gamma Glutamyl Transferase	54	< 60
Inflammatory Markers		
ESR	102	< 20 mm/ hr
CRP	10	< 6 mg/L
D-Dimer	2.0	< 0.5 µg/mL

In view of the abdominal pain, contrast-enhanced computed tomography of the abdomen was performed. The results showed multiple enlarged necrotic conglomerate mesenteric and perigastric regional lymph nodes, diffuse omental and peritoneal nodularity with irregular thickening, and mild ascites. Small bowel loops appeared slightly matted. Imaging features were suggestive of tuberculous peritonitis. A mediastinal window incidentally showed evidence of PTE, so a computed tomography pulmonary angiogram (CTPA) was planned.

**Figure 1:**
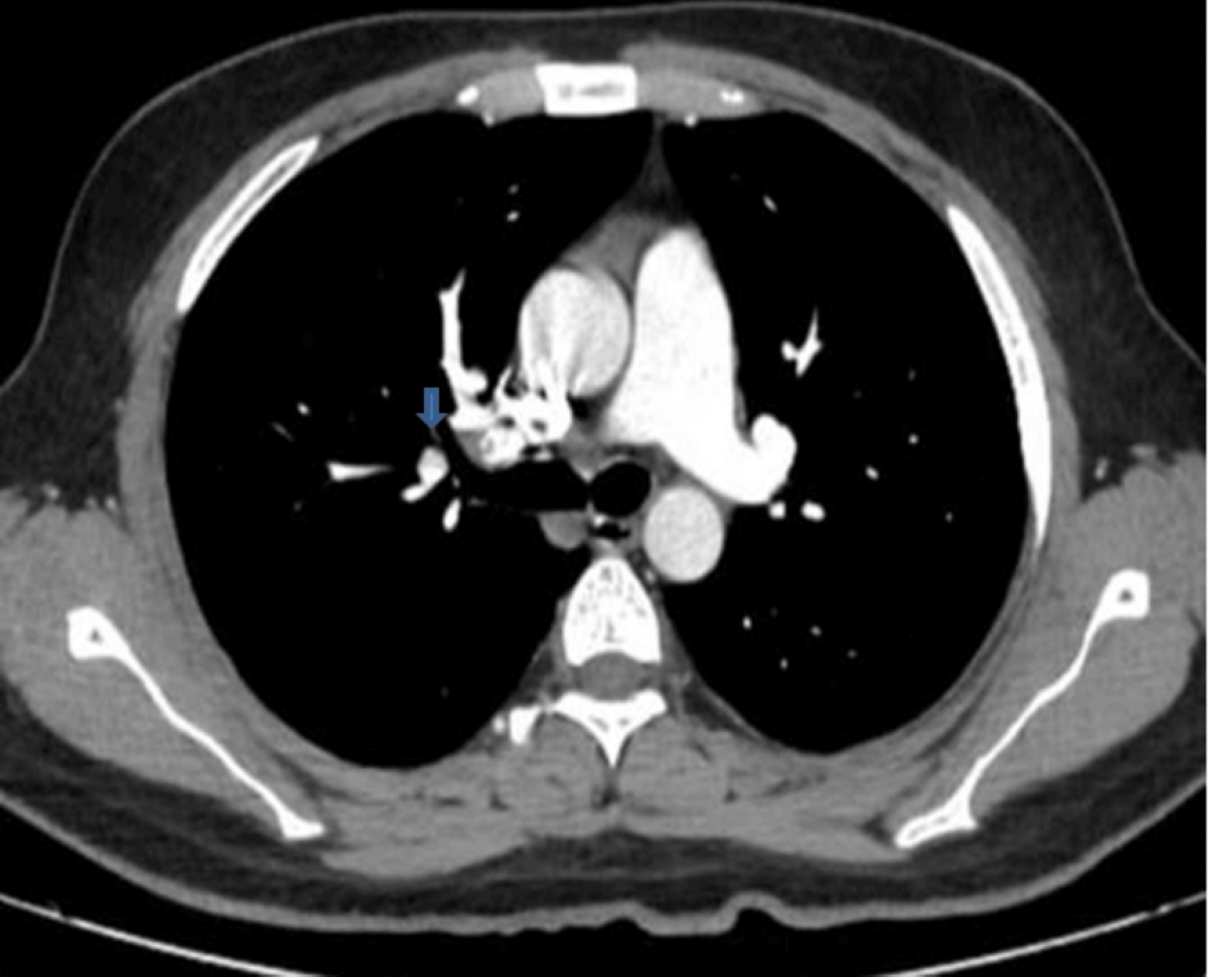
Computed tomography pulmonary angiogram showing thrombus in the right distal pulmonary artery

The CTPA (Figure 1) revealed thrombosis of the distal right main pulmonary artery extending into the right upper lobar and interlobular arteries along with subsegmental branches of the right lower lobe pulmonary artery. The patient was started on intravenous unfractionated heparin. Complement levels (C3, C4) and the thrombophilia profile were negative.

An omental biopsy was performed and sent for GeneXpert and histopathological examination. Histopathology showed necrotizing granulomatous inflammation. The AFB smear and GeneXpert results were positive for *Mycobacterium tuberculosis* and showed rifampicin sensitivity. The patient was started on anti-tubercular treatment (ATT), with full doses of isoniazid, rifampicin, pyrazinamide, and ethambutol. He was subsequently transitioned from intravenous heparin to a novel oral anticoagulant.

In view of the persistent fever spikes, blood and urine cultures were sent. Blood culture grew *Staphylococcus epidermidis*, and clindamycin was added based on sensitivity. Urine culture grew *Escherichia coli* sensitive to piperacillin-tazobactam, which was also initiated. 

**Figure 2:**
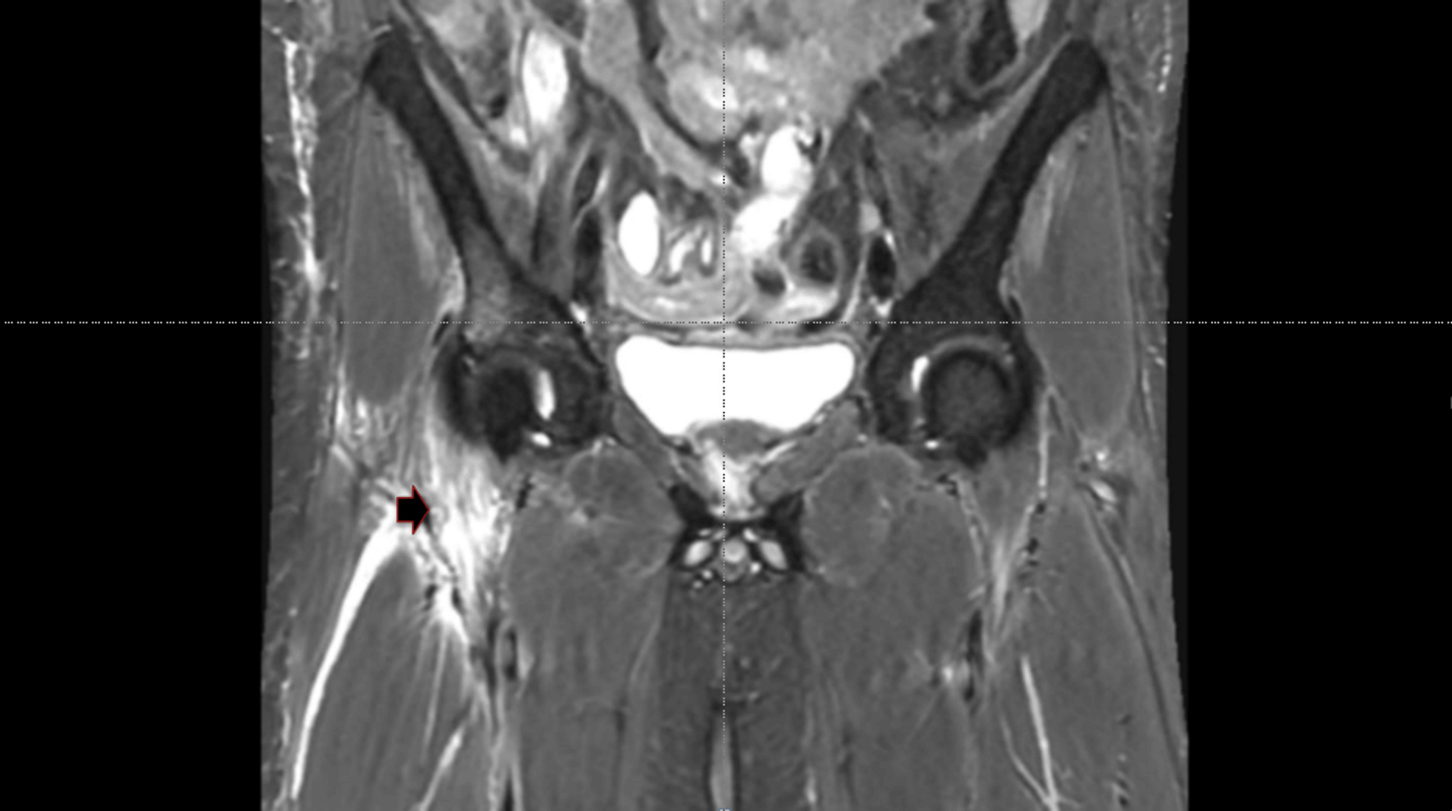
Magnetic resonance imaging of the pelvis showing a right anterior superior iliac spine abscess

The patient developed a swelling measuring 2 × 3 cm in the right inguinal region, which had been present for the past two weeks. Magnetic resonance imaging of the pelvis (Figure 2) showed osteomyelitis of the right anterior superior iliac spine and acetabulum with an associated intramuscular abscess. Ultrasound-guided aspiration of the abscess was performed, and the sample was sent for GeneXpert, AFB smear, and bacterial culture and sensitivity. The AFB smear and GeneXpert results were positive for *Mycobacterium tuberculosis *and showed rifampicin sensitivity. Bacterial culture showed no growth. The patient was managed in the ward and discharged on day 15. At the one-month follow-up, he had gained weight (3 kg) and resumed work in his hometown.

## Discussion

The occurrence of PTE in patients with TB can be attributed to the inflammatory state induced by active infection. TB contributes to a hypercoagulable state through a triad of mechanisms: systemic inflammation, venous stasis, and endothelial dysfunction. Pro-inflammatory cytokines, such as IL-1, IL-6, and TNF-α, are produced during the acute phase of TB. These cytokines stimulate hepatocytes to produce acute-phase reactants and procoagulant factors, thereby contributing to thrombogenesis. Studies have shown that patients with active TB often exhibit thrombocytosis and elevated levels of fibrinogen, factor VIII, and plasminogen activator inhibitor-1. By contrast, natural anticoagulants such as antithrombin III and protein C are reduced, further promoting a hypercoagulable milieu [5].

In this case, the patient presented with disseminated TB and developed PTE, manifesting with high-grade fever, abdominal pain, vomiting, loss of appetite, and significant weight loss. Notably, there was no clinical or radiological evidence of DVT. Such a presentation, in which PTE occurs without detectable DVT, is referred to as DNPE, which has been associated with a more favorable prognosis than PTE associated with DVT. By contrast, the presence of concurrent DVT may indicate a greater thrombotic burden and is described as a poor prognostic factor in some studies.

The early initiation of ATT has been shown to reduce inflammation and mitigate the hypercoagulable state associated with active TB [6]. Notably, however, ATT, particularly rifampicin, may independently increase the risk of PTE because of its potential to cause coagulation abnormalities [6].

## Conclusions

Patients with severe or disseminated TB are at increased risk of developing PTE. Clinicians should be vigilant in recognizing this association, and PTE risk assessment should be considered soon after the diagnosis of TB is confirmed. TB, as a chronic inflammatory disease, activates the coagulation cascade, resulting in thrombin generation and promoting thrombotic events. The early and appropriate initiation of anti-TB treatment plays a dual role, not only addressing the underlying infection but also helping reduce the inflammatory and prothrombotic burden, thereby potentially lowering the risk of PTE.
